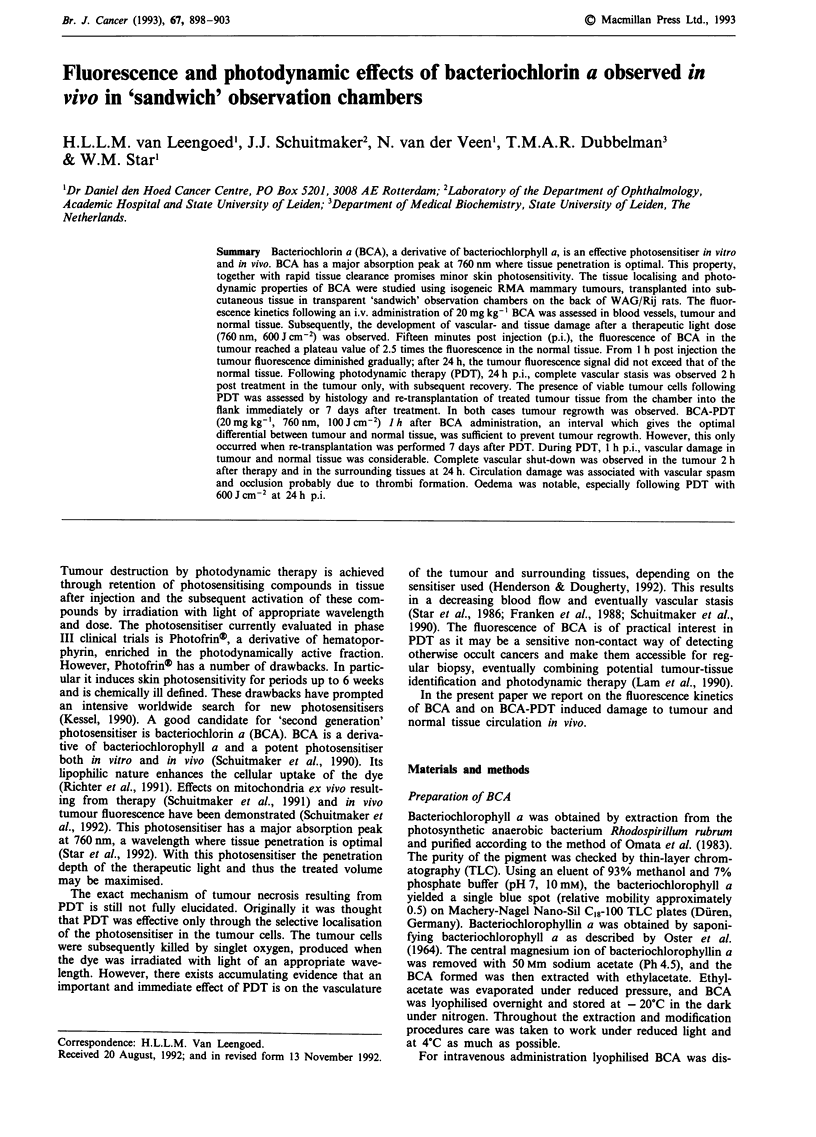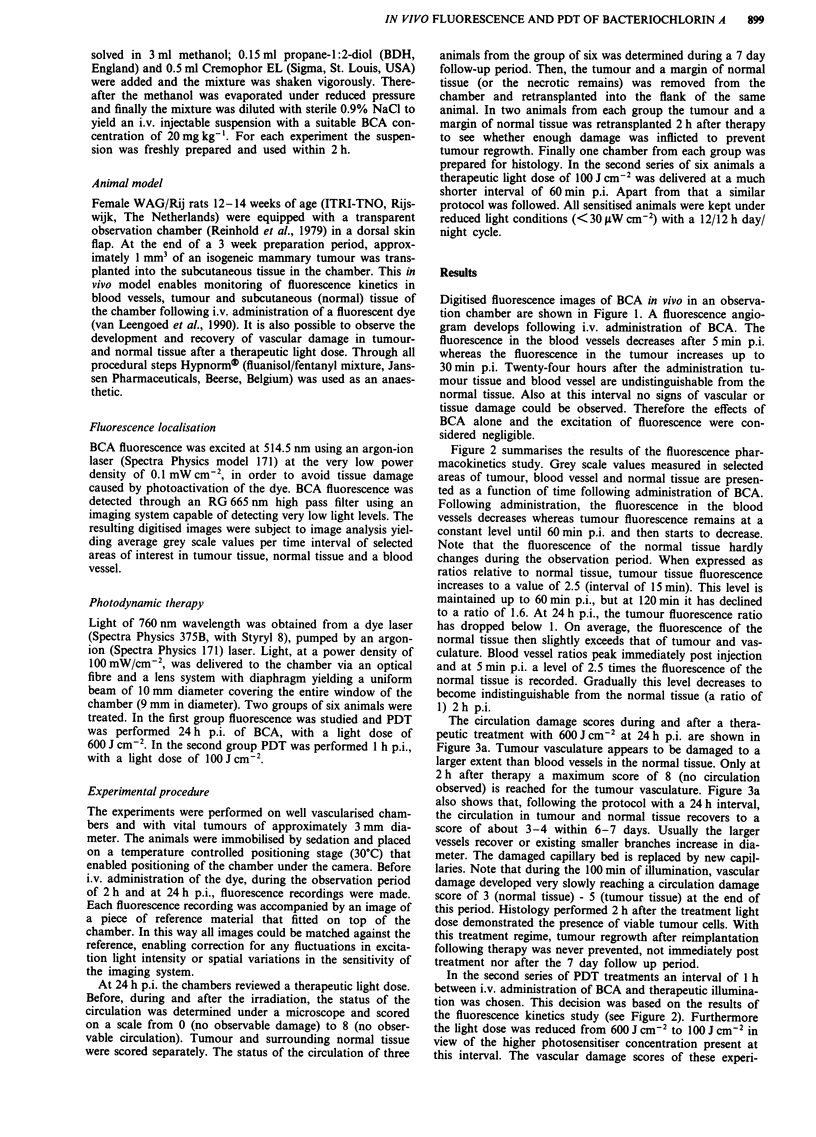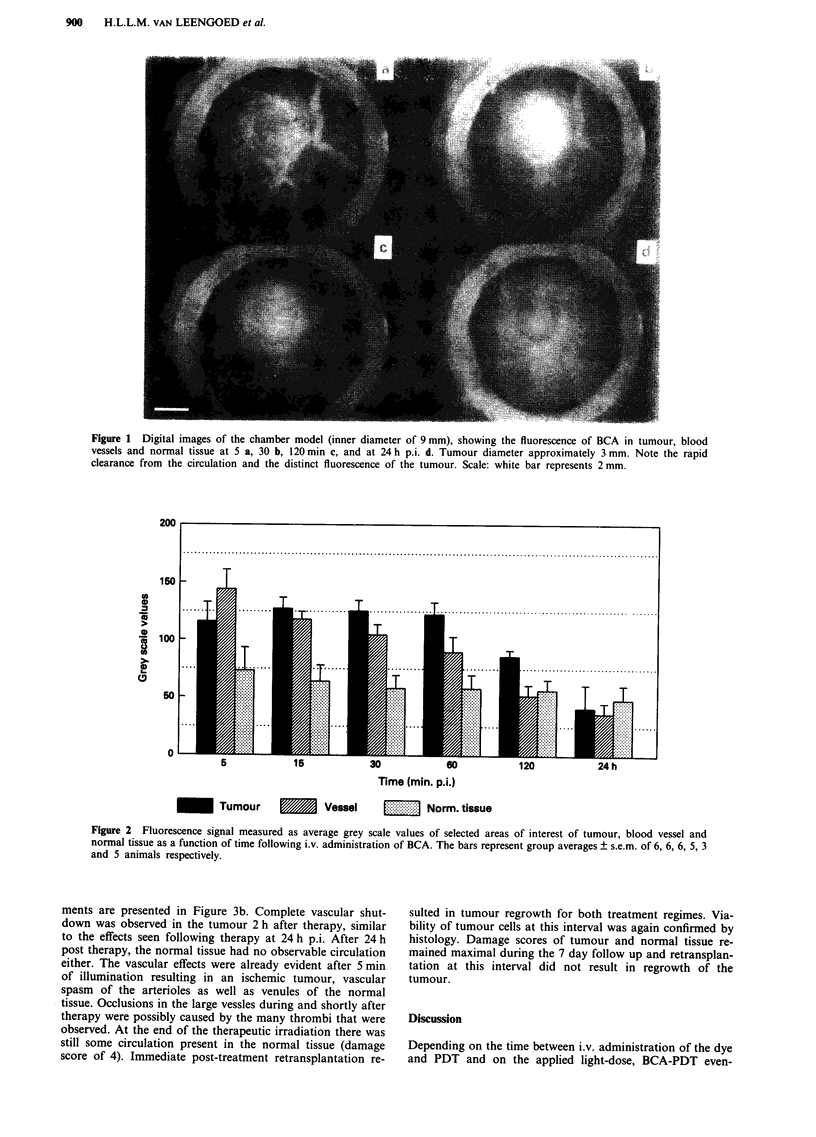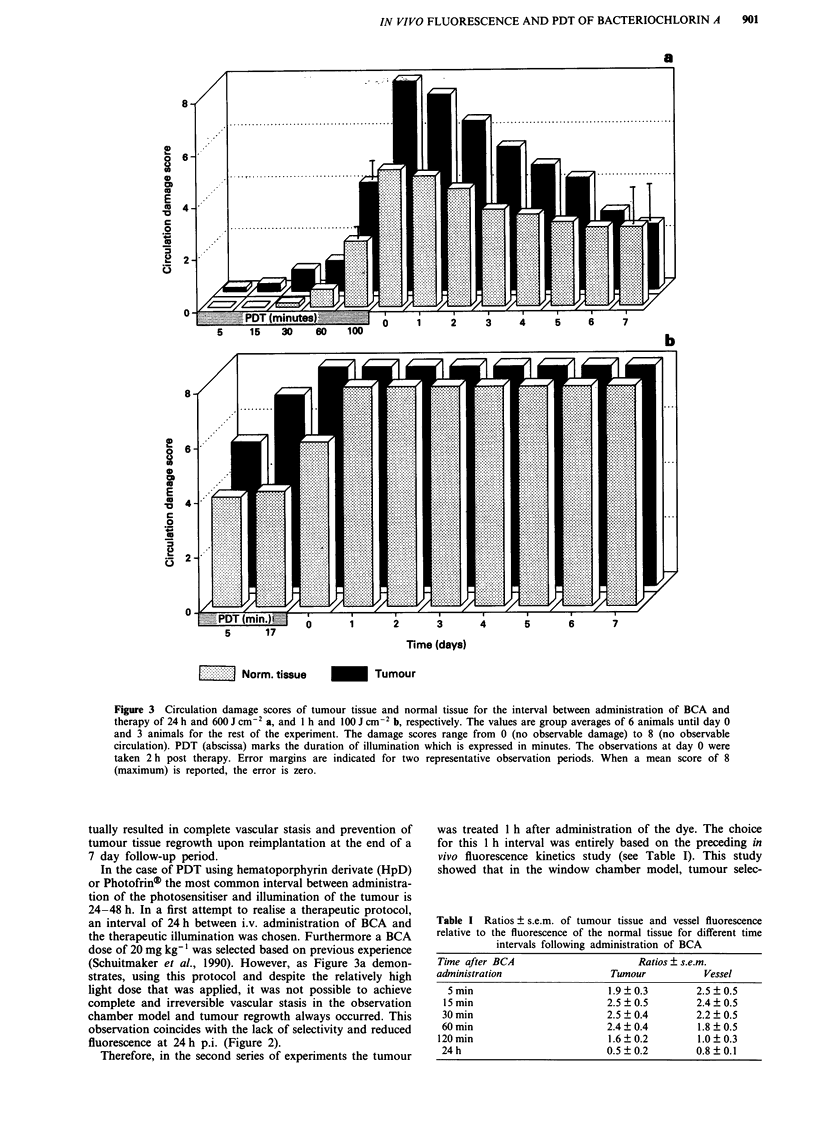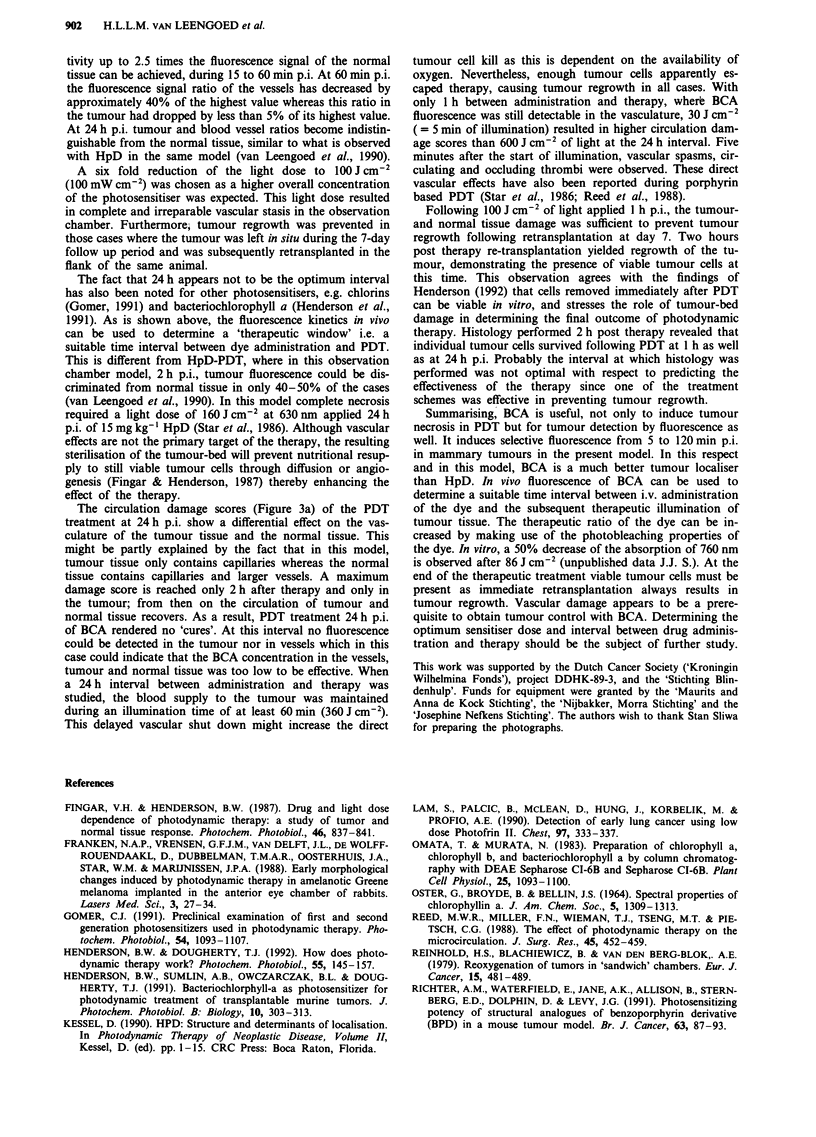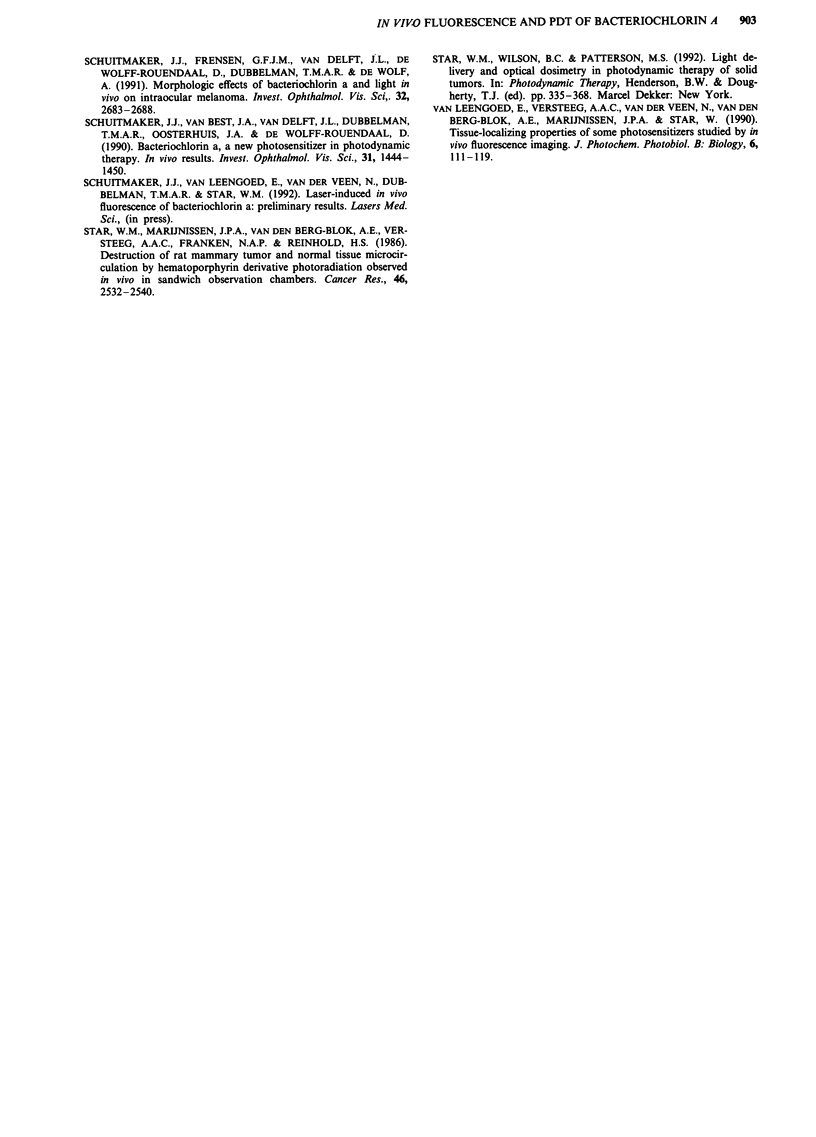# Fluorescence and photodynamic effects of bacteriochlorin a observed in vivo in 'sandwich' observation chambers.

**DOI:** 10.1038/bjc.1993.168

**Published:** 1993-05

**Authors:** H. L. van Leengoed, J. J. Schuitmaker, N. van der Veen, T. M. Dubbelman, W. M. Star

**Affiliations:** Dr Daniel den Hoed Cancer Centre, Rotterdam, The Netherlands.

## Abstract

**Images:**


*Br. J. Cancer* (1993), **67**, 898–903 © Macmillan Press Ltd., 1993

### Fluorescence and photodynamic effects of bacteriochlorin *a* observed *in vivo* in 'sandwich' observation chambers

H.L.L.M. van Leengoed[1], J.J. Schuitmaker[2], N. van der Veen[1], T.M.A.R. Dubbelman[3] & W.M. Star[1]

[1]*Dr Daniel den Hoed Cancer Centre, PO Box 5201, 3008 AE Rotterdam;* [2]*Laboratory of the Department of Ophthalmology, Academic Hospital and State University of Leiden;* [3]*Department of Medical Biochemistry, State University of Leiden, The Netherlands.*

**Summary** Bacteriochlorin *a* (BCA), a derivative of bacteriochlorphyll *a*, is an effective photosensitiser *in vitro* and *in vivo*. BCA has a major absorption peak at 760 nm where tissue penetration is optimal. This property, together with rapid tissue clearance promises minor skin photosensitivity. The tissue localising and photodynamic properties of BCA were studied using isogeneic RMA mammary tumours, transplanted into subcutaneous tissue in transparent 'sandwich' observation chambers on the back of WAG/Rij rats. The fluorescence kinetics following an i.v. administration of 20 mg kg$^{-1}$ BCA was assessed in blood vessels, tumour and normal tissue. Subsequently, the development of vascular- and tissue damage after a therapeutic light dose (760 nm, 600 J cm$^{-2}$) was observed. Fifteen minutes post injection (p.i.), the fluorescence of BCA in the tumour reached a plateau value of 2.5 times the fluorescence in the normal tissue. From 1 h post injection the tumour fluorescence diminished gradually; after 24 h, the tumour fluorescence signal did not exceed that of the normal tissue. Following photodynamic therapy (PDT), 24 h p.i., complete vascular stasis was observed 2 h post treatment in the tumour only, with subsequent recovery. The presence of viable tumour cells following PDT was assessed by histology and re-transplantation of treated tumour tissue from the chamber into the flank immediately or 7 days after treatment. In both cases tumour regrowth was observed. BCA-PDT (20 mg kg$^{-1}$, 760 nm, 100 J cm$^{-2}$) *1 h* after BCA administration, an interval which gives the optimal differential between tumour and normal tissue, was sufficient to prevent tumour regrowth. However, this only occurred when re-transplantation was performed 7 days after PDT. During PDT, 1 h p.i., vascular damage in tumour and normal tissue was considerable. Complete vascular shut-down was observed in the tumour 2 h after therapy and in the surrounding tissues at 24 h. Circulation damage was associated with vascular spasm and occlusion probably due to thrombi formation. Oedema was notable, especially following PDT with 600 J cm$^{-2}$ at 24 h p.i.

Tumour destruction by photodynamic therapy is achieved through retention of photosensitising compounds in tissue after injection and the subsequent activation of these compounds by irradiation with light of appropriate wavelength and dose. The photosensitiser currently evaluated in phase III clinical trials is Photofrin®, a derivative of hematoporphyrin, enriched in the photodynamically active fraction. However, Photofrin® has a number of drawbacks. In particular it induces skin photosensitivity for periods up to 6 weeks and is chemically ill defined. These drawbacks have prompted an intensive worldwide search for new photosensitisers (Kessel, 1990). A good candidate for 'second generation' photosensitiser is bacteriochlorin *a* (BCA). BCA is a derivative of bacteriochlorophyll *a* and a potent photosensitiser both *in vitro* and *in vivo* (Schuitmaker *et al.*, 1990). Its lipophilic nature enhances the cellular uptake of the dye (Richter *et al.*, 1991). Effects on mitochondria *ex vivo* resulting from therapy (Schuitmaker *et al.*, 1991) and *in vivo* tumour fluorescence have been demonstrated (Schuitmaker *et al.*, 1992). This photosensitiser has a major absorption peak at 760 nm, a wavelength where tissue penetration is optimal (Star *et al.*, 1992). With this photosensitiser the penetration depth of the therapeutic light and thus the treated volume may be maximised.

The exact mechanism of tumour necrosis resulting from PDT is still not fully elucidated. Originally it was thought that PDT was effective only through the selective localisation of the photosensitiser in the tumour cells. The tumour cells were subsequently killed by singlet oxygen, produced when the dye was irradiated with light of an appropriate wavelength. However, there exists accumulating evidence that an important and immediate effect of PDT is on the vasculature of the tumour and surrounding tissues, depending on the sensitiser used (Henderson & Dougherty, 1992). This results in a decreasing blood flow and eventually vascular stasis (Star *et al.*, 1986; Franken *et al.*, 1988; Schuitmaker *et al.*, 1990). The fluorescence of BCA is of practical interest in PDT as it may be a sensitive non-contact way of detecting otherwise occult cancers and make them accessible for regular biopsy, eventually combining potential tumour-tissue identification and photodynamic therapy (Lam *et al.*, 1990).

In the present paper we report on the fluorescence kinetics of BCA and on BCA-PDT induced damage to tumour and normal tissue circulation *in vivo*.

### Materials and methods

### Preparation of BCA

Bacteriochlorophyll *a* was obtained by extraction from the photosynthetic anaerobic bacterium *Rhodospirillum rubrum* and purified according to the method of Omata *et al.* (1983). The purity of the pigment was checked by thin-layer chromatography (TLC). Using an eluent of 93% methanol and 7% phosphate buffer (pH 7, 10 mM), the bacteriochlorophyll *a* yielded a single blue spot (relative mobility approximately 0.5) on Machery-Nagel Nano-Sil $C_{18}$-100 TLC plates (Düren, Germany). Bacteriochlorophyllin *a* was obtained by saponifying bacteriochlorophyll *a* as described by Oster *et al.* (1964). The central magnesium ion of bacteriochlorophyllin *a* was removed with 50 Mm sodium acetate (Ph 4.5), and the BCA formed was then extracted with ethylacetate. Ethylacetate was evaporated under reduced pressure, and BCA was lyophilised overnight and stored at – 20°C in the dark under nitrogen. Throughout the extraction and modification procedures care was taken to work under reduced light and at 4°C as much as possible.

For intravenous administration lyophilised BCA was dis-

Correspondence: H.L.L.M. Van Leengoed.
Received 20 August, 1992; and in revised form 13 November 1992.

solved in 3 ml methanol; 0.15 ml propane-1:2-diol (BDH, England) and 0.5 ml Cremophor EL (Sigma, St. Louis, USA) were added and the mixture was shaken vigorously. Thereafter the methanol was evaporated under reduced pressure and finally the mixture was diluted with sterile 0.9% NaCl to yield an i.v. injectable suspension with a suitable BCA concentration of 20 mg $kg^{-1}$. For each experiment the suspension was freshly prepared and used within 2 h.

### *Animal model*

Female WAG/Rij rats 12–14 weeks of age (ITRI-TNO, Rijswijk, The Netherlands) were equipped with a transparent observation chamber (Reinhold *et al.*, 1979) in a dorsal skin flap. At the end of a 3 week preparation period, approximately 1 $mm^3$ of an isogeneic mammary tumour was transplanted into the subcutaneous tissue in the chamber. This *in vivo* model enables monitoring of fluorescence kinetics in blood vessels, tumour and subcutaneous (normal) tissue of the chamber following i.v. administration of a fluorescent dye (van Leengoed *et al.*, 1990). It is also possible to observe the development and recovery of vascular damage in tumour- and normal tissue after a therapeutic light dose. Through all procedural steps Hypnorm® (fluanisol/fentanyl mixture, Janssen Pharmaceuticals, Beerse, Belgium) was used as an anaesthetic.

### *Fluorescence localisation*

BCA fluorescence was excited at 514.5 nm using an argon-ion laser (Spectra Physics model 171) at the very low power density of 0.1 mW $cm^{-2}$, in order to avoid tissue damage caused by photoactivation of the dye. BCA fluorescence was detected through an RG 665 nm high pass filter using an imaging system capable of detecting very low light levels. The resulting digitised images were subject to image analysis yielding average grey scale values per time interval of selected areas of interest in tumour tissue, normal tissue and a blood vessel.

### *Photodynamic therapy*

Light of 760 nm wavelength was obtained from a dye laser (Spectra Physics 375B, with Styryl 8), pumped by an argon-ion (Spectra Physics 171) laser. Light, at a power density of 100 mW/$cm^{-2}$, was delivered to the chamber via an optical fibre and a lens system with diaphragm yielding a uniform beam of 10 mm diameter covering the entire window of the chamber (9 mm in diameter). Two groups of six animals were treated. In the first group fluorescence was studied and PDT was performed 24 h p.i. of BCA, with a light dose of 600 J $cm^{-2}$. In the second group PDT was performed 1 h p.i., with a light dose of 100 J $cm^{-2}$.

### *Experimental procedure*

The experiments were performed on well vascularised chambers and with vital tumours of approximately 3 mm diameter. The animals were immobilised by sedation and placed on a temperature controlled positioning stage (30°C) that enabled positioning of the chamber under the camera. Before i.v. administration of the dye, during the observation period of 2 h and at 24 h p.i., fluorescence recordings were made. Each fluorescence recording was accompanied by an image of a piece of reference material that fitted on top of the chamber. In this way all images could be matched against the reference, enabling correction for any fluctuations in excitation light intensity or spatial variations in the sensitivity of the imaging system.

At 24 h p.i. the chambers reviewed a therapeutic light dose. Before, during and after the irradiation, the status of the circulation was determined under a microscope and scored on a scale from 0 (no observable damage) to 8 (no observable circulation). Tumour and surrounding normal tissue were scored separately. The status of the circulation of three animals from the group of six was determined during a 7 day follow-up period. Then, the tumour and a margin of normal tissue (or the necrotic remains) was removed from the chamber and retransplanted into the flank of the same animal. In two animals from each group the tumour and a margin of normal tissue was retransplanted 2 h after therapy to see whether enough damage was inflicted to prevent tumour regrowth. Finally one chamber from each group was prepared for histology. In the second series of six animals a therapeutic light dose of 100 J $cm^{-2}$ was delivered at a much shorter interval of 60 min p.i. Apart from that a similar protocol was followed. All sensitised animals were kept under reduced light conditions ($<30\,\mu W\,cm^{-2}$) with a 12/12 h day/night cycle.

### Results

Digitised fluorescence images of BCA *in vivo* in an observation chamber are shown in Figure 1. A fluorescence angiogram develops following i.v. administration of BCA. The fluorescence in the blood vessels decreases after 5 min p.i. whereas the fluorescence in the tumour increases up to 30 min p.i. Twenty-four hours after the administration tumour tissue and blood vessel are undistinguishable from the normal tissue. Also at this interval no signs of vascular or tissue damage could be observed. Therefore the effects of BCA alone and the excitation of fluorescence were considered negligible.

Figure 2 summarises the results of the fluorescence pharmacokinetics study. Grey scale values measured in selected areas of tumour, blood vessel and normal tissue are presented as a function of time following administration of BCA. Following administration, the fluorescence in the blood vessels decreases whereas tumour fluorescence remains at a constant level until 60 min p.i. and then starts to decrease. Note that the fluorescence of the normal tissue hardly changes during the observation period. When expressed as ratios relative to normal tissue, tumour tissue fluorescence increases to a value of 2.5 (interval of 15 min). This level is maintained up to 60 min p.i., but at 120 min it has declined to a ratio of 1.6. At 24 h p.i., the tumour fluorescence ratio has dropped below 1. On average, the fluorescence of the normal tissue then slightly exceeds that of tumour and vasculature. Blood vessel ratios peak immediately post injection and at 5 min p.i. a level of 2.5 times the fluorescence of the normal tissue is recorded. Gradually this level decreases to become indistinguishable from the normal tissue (a ratio of 1) 2 h p.i.

The circulation damage scores during and after a therapeutic treatment with 600 J $cm^{-2}$ at 24 h p.i. are shown in Figure 3a. Tumour vasculature appears to be damaged to a larger extent than blood vessels in the normal tissue. Only at 2 h after therapy a maximum score of 8 (no circulation observed) is reached for the tumour vasculature. Figure 3a also shows that, following the protocol with a 24 h interval, the circulation in tumour and normal tissue recovers to a score of about 3–4 within 6–7 days. Usually the larger vessels recover or existing smaller branches increase in diameter. The damaged capillary bed is replaced by new capillaries. Note that during the 100 min of illumination, vascular damage developed very slowly reaching a circulation damage score of 3 (normal tissue) - 5 (tumour tissue) at the end of this period. Histology performed 2 h after the treatment light dose demonstrated the presence of viable tumour cells. With this treatment regime, tumour regrowth after reimplantation following therapy was never prevented, not immediately post treatment nor after the 7 day follow up period.

In the second series of PDT treatments an interval of 1 h between i.v. administration of BCA and therapeutic illumination was chosen. This decision was based on the results of the fluorescence kinetics study (see Figure 2). Furthermore the light dose was reduced from 600 J $cm^{-2}$ to 100 J $cm^{-2}$ in view of the higher photosensitiser concentration present at this interval. The vascular damage scores of these experi-

**Figure 1** Digital images of the chamber model (inner diameter of 9 mm), showing the fluorescence of BCA in tumour, blood vessels and normal tissue at 5 **a**, 30 **b**, 120 min **c**, and at 24 h p.i. **d**. Tumour diameter approximately 3 mm. Note the rapid clearance from the circulation and the distinct fluorescence of the tumour. Scale: white bar represents 2 mm.

**Figure 2** Fluorescence signal measured as average grey scale values of selected areas of interest of tumour, blood vessel and normal tissue as a function of time following i.v. administration of BCA. The bars represent group averages ± s.e.m. of 6, 6, 6, 5, 3 and 5 animals respectively.

ments are presented in Figure 3b. Complete vascular shutdown was observed in the tumour 2 h after therapy, similar to the effects seen following therapy at 24 h p.i. After 24 h post therapy, the normal tissue had no observable circulation either. The vascular effects were already evident after 5 min of illumination resulting in an ischemic tumour, vascular spasm of the arterioles as well as venules of the normal tissue. Occlusions in the large vessles during and shortly after therapy were possibly caused by the many thrombi that were observed. At the end of the therapeutic irradiation there was still some circulation present in the normal tissue (damage score of 4). Immediate post-treatment retransplantation resulted in tumour regrowth for both treatment regimes. Viability of tumour cells at this interval was again confirmed by histology. Damage scores of tumour and normal tissue remained maximal during the 7 day follow up and retransplantation at this interval did not result in regrowth of the tumour.

### Discussion

Depending on the time between i.v. administration of the dye and PDT and on the applied light-dose, BCA-PDT even-

**Figure 3** Circulation damage scores of tumour tissue and normal tissue for the interval between administration of BCA and therapy of 24 h and 600 J $cm^{-2}$ **a**, and 1 h and 100 J $cm^{-2}$ **b**, respectively. The values are group averages of 6 animals until day 0 and 3 animals for the rest of the experiment. The damage scores range from 0 (no observable damage) to 8 (no observable circulation). PDT (abscissa) marks the duration of illumination which is expressed in minutes. The observations at day 0 were taken 2 h post therapy. Error margins are indicated for two representative observation periods. When a mean score of 8 (maximum) is reported, the error is zero.

tually resulted in complete vascular stasis and prevention of tumour tissue regrowth upon reimplantation at the end of a 7 day follow-up period.

In the case of PDT using hematoporphyrin derivate (HpD) or Photofrin® the most common interval between administration of the photosensitiser and illumination of the tumour is 24–48 h. In a first attempt to realise a therapeutic protocol, an interval of 24 h between i.v. administration of BCA and the therapeutic illumination was chosen. Furthermore a BCA dose of 20 mg $kg^{-1}$ was selected based on previous experience (Schuitmaker *et al.*, 1990). However, as Figure 3a demonstrates, using this protocol and despite the relatively high light dose that was applied, it was not possible to achieve complete and irreversible vascular stasis in the observation chamber model and tumour regrowth always occurred. This observation coincides with the lack of selectivity and reduced fluorescence at 24 h p.i. (Figure 2).

Therefore, in the second series of experiments the tumour was treated 1 h after administration of the dye. The choice for this 1 h interval was entirely based on the preceding *in vivo* fluorescence kinetics study (see Table I). This study showed that in the window chamber model, tumour selec-

**Table I** Ratios ± s.e.m. of tumour tissue and vessel fluorescence relative to the fluorescence of the normal tissue for different time intervals following administration of BCA

| *Time after BCA administration* | *Ratios ± s.e.m.* *Tumour* | *Vessel* |
|---|---|---|
| 5 min | 1.9 ± 0.3 | 2.5 ± 0.5 |
| 15 min | 2.5 ± 0.5 | 2.4 ± 0.5 |
| 30 min | 2.5 ± 0.4 | 2.2 ± 0.5 |
| 60 min | 2.4 ± 0.4 | 1.8 ± 0.5 |
| 120 min | 1.6 ± 0.2 | 1.0 ± 0.3 |
| 24 h | 0.5 ± 0.2 | 0.8 ± 0.1 |

tivity up to 2.5 times the fluorescence signal of the normal tissue can be achieved, during 15 to 60 min p.i. At 60 min p.i. the fluorescence signal ratio of the vessels has decreased by approximately 40% of the highest value whereas this ratio in the tumour had dropped by less than 5% of its highest value. At 24 h p.i. tumour and blood vessel ratios become indistinguishable from the normal tissue, similar to what is observed with HpD in the same model (van Leengoed *et al.*, 1990).

A six fold reduction of the light dose to 100 J $cm^{-2}$ (100 mW $cm^{-2}$) was chosen as a higher overall concentration of the photosensitiser was expected. This light dose resulted in complete and irreparable vascular stasis in the observation chamber. Furthermore, tumour regrowth was prevented in those cases where the tumour was left *in situ* during the 7-day follow up period and was subsequently retransplanted in the flank of the same animal.

The fact that 24 h appears not to be the optimum interval has also been noted for other photosensitisers, e.g. chlorins (Gomer, 1991) and bacteriochlorophyll *a* (Henderson *et al.*, 1991). As is shown above, the fluorescence kinetics *in vivo* can be used to determine a 'therapeutic window' i.e. a suitable time interval between dye administration and PDT. This is different from HpD-PDT, where in this observation chamber model, 2 h p.i., tumour fluorescence could be discriminated from normal tissue in only 40–50% of the cases (van Leengoed *et al.*, 1990). In this model complete necrosis required a light dose of 160 J $cm^{-2}$ at 630 nm applied 24 h p.i. of 15 mg $kg^{-1}$ HpD (Star *et al.*, 1986). Although vascular effects are not the primary target of the therapy, the resulting sterilisation of the tumour-bed will prevent nutritional resupply to still viable tumour cells through diffusion or angiogenesis (Fingar & Henderson, 1987) thereby enhancing the effect of the therapy.

The circulation damage scores (Figure 3a) of the PDT treatment at 24 h p.i. show a differential effect on the vasculature of the tumour tissue and the normal tissue. This might be partly explained by the fact that in this model, tumour tissue only contains capillaries whereas the normal tissue contains capillaries and larger vessels. A maximum damage score is reached only 2 h after therapy and only in the tumour; from then on the circulation of tumour and normal tissue recovers. As a result, PDT treatment 24 h p.i. of BCA rendered no 'cures'. At this interval no fluorescence could be detected in the tumour nor in vessels which in this case could indicate that the BCA concentration in the vessels, tumour and normal tissue was too low to be effective. When a 24 h interval between administration and therapy was studied, the blood supply to the tumour was maintained during an illumination time of at least 60 min (360 J $cm^{-2}$). This delayed vascular shut down might increase the direct tumour cell kill as this is dependent on the availability of oxygen. Nevertheless, enough tumour cells apparently escaped therapy, causing tumour regrowth in all cases. With only 1 h between administration and therapy, where BCA fluorescence was still detectable in the vasculature, 30 J $cm^{-2}$ ( = 5 min of illumination) resulted in higher circulation damage scores than 600 J $cm^{-2}$ of light at the 24 h interval. Five minutes after the start of illumination, vascular spasms, circulating and occluding thrombi were observed. These direct vascular effects have also been reported during porphyrin based PDT (Star *et al.*, 1986; Reed *et al.*, 1988).

Following 100 J $cm^{-2}$ of light applied 1 h p.i., the tumour- and normal tissue damage was sufficient to prevent tumour regrowth following retransplantation at day 7. Two hours post therapy re-transplantation yielded regrowth of the tumour, demonstrating the presence of viable tumour cells at this time. This observation agrees with the findings of Henderson (1992) that cells removed immediately after PDT can be viable *in vitro*, and stresses the role of tumour-bed damage in determining the final outcome of photodynamic therapy. Histology performed 2 h post therapy revealed that individual tumour cells survived following PDT at 1 h as well as at 24 h p.i. Probably the interval at which histology was performed was not optimal with respect to predicting the effectiveness of the therapy since one of the treatment schemes was effective in preventing tumour regrowth.

Summarising, BCA is useful, not only to induce tumour necrosis in PDT but for tumour detection by fluorescence as well. It induces selective fluorescence from 5 to 120 min p.i. in mammary tumours in the present model. In this respect and in this model, BCA is a much better tumour localiser than HpD. *In vivo* fluorescence of BCA can be used to determine a suitable time interval between i.v. administration of the dye and the subsequent therapeutic illumination of tumour tissue. The therapeutic ratio of the dye can be increased by making use of the photobleaching properties of the dye. *In vitro*, a 50% decrease of the absorption of 760 nm is observed after 86 J $cm^{-2}$ (unpublished data J.J. S.). At the end of the therapeutic treatment viable tumour cells must be present as immediate retransplantation always results in tumour regrowth. Vascular damage appears to be a prerequisite to obtain tumour control with BCA. Determining the optimum sensitiser dose and interval between drug administration and therapy should be the subject of further study.

This work was supported by the Dutch Cancer Society ('Kroningin Wilhelmina Fonds'), project DDHK-89-3, and the 'Stichting Blindenhulp'. Funds for equipment were granted by the 'Maurits and Anna de Kock Stichting', the 'Nijbakker, Morra Stichting' and the 'Josephine Nefkens Stichting'. The authors wish to thank Stan Sliwa for preparing the photographs.

### References

FINGAR, V.H. & HENDERSON, B.W. (1987). Drug and light dose dependence of photodynamic therapy: a study of tumor and normal tissue response. *Photochem. Photobiol.*, **46**, 837–841.

FRANKEN, N.A.P., VRENSEN, G.F.J.M., VAN DELFT, J.L., DE WOLFF-ROUENDAAKL, D., DUBBELMAN, T.M.A.R., OOSTERHUIS, J.A., STAR, W.M. & MARIJNISSEN, J.P.A. (1988). Early morphological changes induced by photodynamic therapy in amelanotic Greene melanoma implanted in the anterior eye chamber of rabbits. *Lasers Med. Sci.*, **3**, 27–34.

GOMER, C.J. (1991). Preclinical examination of first and second generation photosensitizers used in photodynamic therapy. *Photochem. Photobiol.*, **54**, 1093–1107.

HENDERSON, B.W. & DOUGHERTY, T.J. (1992). How does photodynamic therapy work? *Photochem. Photobiol.*, **55**, 145–157.

HENDERSON, B.W., SUMLIN, A.B., OWCZARCZAK, B.L. & DOUGHERTY, T.J. (1991). Bacteriochlorphyll-a as photosensitizer for photodynamic treatment of transplantable murine tumors. *J. Photochem. Photobiol. B: Biology*, **10**, 303–313.

KESSEL, D. (1990). HPD: Structure and determinants of localisation. In *Photodynamic Therapy of Neoplastic Disease, Volume II*, Kessel, D. (ed). pp. 1–15. CRC Press: Boca Raton, Florida.

LAM, S., PALCIC, B., MCLEAN, D., HUNG, J., KORBELIK, M. & PROFIO, A.E. (1990). Detection of early lung cancer using low dose Photofrin II. *Chest*, **97**, 333–337.

OMATA, T. & MURATA, N. (1983). Preparation of chlorophyll a, chlorophyll b, and bacteriochlorophyll a by column chromatography with DEAE Sepharose CI-6B and Sepharose CI-6B. *Plant Cell Physiol.*, **25**, 1093–1100.

OSTER, G., BROYDE, B. & BELLIN, J.S. (1964). Spectral properties of chlorophyllin a. *J. Am. Chem. Soc.*, **5**, 1309–1313.

REED, M.W.R., MILLER, F.N., WIEMAN, T.J., TSENG, M.T. & PIETSCH, C.G. (1988). The effect of photodynamic therapy on the microcirculation. *J. Surg. Res.*, **45**, 452–459.

REINHOLD, H.S., BLACHIEWICZ, B. & VAN DEN BERG-BLOK,. A.E. (1979). Reoxygenation of tumors in 'sandwich' chambers. *Eur. J. Cancer*, **15**, 481–489.

RICHTER, A.M., WATERFIELD, E., JANE, A.K., ALLISON, B., STERNBERG, E.D., DOLPHIN, D. & LEVY, J.G. (1991). Photosensitizing potency of structural analogues of benzoporphyrin derivative (BPD) in a mouse tumour model. *Br. J. Cancer*, **63**, 87–93.

SCHUITMAKER, J.J., FRENSEN, G.F.J.M., VAN DELFT, J.L., DE WOLFF-ROUENDAAL, D., DUBBELMAN, T.M.A.R. & DE WOLF, A. (1991). Morphologic effects of bacteriochlorin a and light *in vivo* on intraocular melanoma. *Invest. Ophthalmol. Vis. Sci,*. **32**, 2683–2688.

SCHUITMAKER, J.J., VAN BEST, J.A., VAN DELFT, J.L., DUBBELMAN, T.M.A.R., OOSTERHUIS, J.A. & DE WOLFF-ROUENDAAL, D. (1990). Bacteriochlorin a, a new photosensitizer in photodynamic therapy. *In vivo* results. *Invest. Ophthalmol. Vis. Sci.*, **31**, 1444–1450.

SCHUITMAKER, J.J., VAN LEENGOED, E., VAN DER VEEN, N., DUBBELMAN, T.M.A.R. & STAR, W.M. (1992). Laser-induced *in vivo* fluorescence of bacteriochlorin a: preliminary results. *Lasers Med. Sci.*, (in press).

STAR, W.M., MARIJNISSEN, J.P.A., VAN DEN BERG-BLOK, A.E., VERSTEEG, A.A.C., FRANKEN, N.A.P. & REINHOLD, H.S. (1986). Destruction of rat mammary tumor and normal tissue microcirculation by hematoporphyrin derivative photoradiation observed *in vivo* in sandwich observation chambers. *Cancer Res.*, **46**, 2532–2540.

STAR, W.M., WILSON, B.C. & PATTERSON, M.S. (1992). Light delivery and optical dosimetry in photodynamic therapy of solid tumors. In: *Photodynamic Therapy*, Henderson, B.W. & Dougherty, T.J. (ed). pp. 335–368. Marcel Dekker: New York.

VAN LEENGOED, E., VERSTEEG, A.A.C., VAN DER VEEN, N., VAN DEN BERG-BLOK, A.E., MARIJNISSEN, J.P.A. & STAR, W. (1990). Tissue-localizing properties of some photosensitizers studied by *in vivo* fluorescence imaging. *J. Photochem. Photobiol. B: Biology*, **6**, 111–119.